# Editorial: Synovial Tissue: Turning the Page to Precision Medicine in Arthritis?

**DOI:** 10.3389/fmed.2021.749062

**Published:** 2021-11-24

**Authors:** Carl Kieran Orr, Frances Humby, João Eurico Fonseca

**Affiliations:** ^1^Centre for Arthritis and Rheumatic Diseases, Saint Vincent's University Hospital, Dublin, Ireland; ^2^Centre for Experimental Medicine and Rheumatology, Queen Mary University of London, London, United Kingdom; ^3^Department of Rheumatology, Mile End Hospital, Barts Health NHS Trust, London, United Kingdom; ^4^Serviço de Reumatologia e Doenças Ósseas Metabólicas, Centro Hospitalar Universitário Lisboa Norte, Centro Académico de Medicina de Lisboa (CAML), Lisbon, Portugal; ^5^Unidade de Investigação em Reumatologia, Instituto de Medicina Molecular, Faculdade de Medicina, Universidade de Lisboa, Centro Académico de Medicina de Lisboa (CAML), Lisbon, Portugal

**Keywords:** synovial biopsy, synovial tissue, synovial tissue analysis, synovial membrane, inflammation, arthritis, precision medicine

It is with great pleasure that we present in this article collection, a timely overview of the rapidly developing field of synovial tissue analysis. Some of the most prominent protagonists in the field have contributed, and the collection walks the reader through everything from the history of the field's development, to technical aspects of sampling, providing an update on the science and clinical applications, as well as discussing potential future perspectives.

A broad consensus exists amongst clinicians and scientists, that a patient-centred, precision medicine approach holds the most promise to improve patient outcomes. The relevance of synovial biopsies in achieving this end is a major theme of this article collection. We are currently at an exciting juncture in this important field. This collection not only discusses the enormous potential of synovial tissue as a research and clinical tool, but also the many challenges in advancing its role in translational and clinical applications. Several key advancements concerning synovial biopsies over the last number of years have together contributed to the rheumatology community discussing in earnest how such sampling can contribute to precision medicine in arthritis.

In the first instance, the technical feasibility, the safety, and the patient tolerability of the procedures used to retrieve the tissue have been extensively studied, and there is now wide acknowledgement that these procedures carry minimal risk and are well-tolerated. Veale provides a historical perspective and overview of synovial biopsy research to date. Four key points emerge, of which arguably the most important concerns the safety of the procedures utilised to sample the synovium; whether ultrasound (US) or arthroscopically guided. Complication rates ranging from 0.4 to 0.9% are reported, and most are not serious. The author outlines the overarching objectives of synovial biopsy research- namely to identify predictors of the development of arthritis, predictors of response to treatment, as well as to objectively measure disease activity and response to treatment. It is widely recognised that realising these objectives will depend on the identification of reliable biomarkers, which will allow for better patient stratification, improved therapies, and the development of new therapeutic targets. Together, these developments will lead to a more precise approach to treatment.

Ingegnoli et al. provide a comprehensive and practical guide to selecting candidates for synovial biopsy. The authors contextualise the current and potential future role for tissue analysis in both the research and the clinical setting. In their contribution, De Bellefon and Lazarou outline the principles underlying US guided biopsy, many of which are immediately relevant to other modalities of synovial biopsy. They provide a comprehensive overview of the two commonly performed US guided techniques, portal and forceps and semi-automatic guillotine biopsy needle. Specific emphasis is placed on peri-procedural anti-sepsis, and they present a step-by step guide, including helpful graphics, for these approaches to sampling. They also present data on some long-debated questions such as assuring the safety of intra-articular steroid after the procedure and the association of lignocaine with chondrolysis.

The technique for US guided biopsy is expanded upon by Polido-Pereira, with a specific focus on medium and large joints, as well as discussing the differences between the two sonographic methodologies. The author points out that it is possible to biopsy nearly all synovial joints. In this review, techniques for biopsying the shoulder, elbow, hip, knee and tibiotalar joint, are described using descriptive anatomy and appropriate visual aids. The author notes that the grade of grayscale synovitis is the most important determinant for synovial tissue yield. It is interesting to note that this review advises that 12 biopsies should be the minimum number to be taken to ensure representative sampling. Others have contested that 6 to 8 might suffice, pointing to evidence that this allows for reliable scores for T-cell infiltration with a variance of <10%, as well as a less than two-fold difference in gene expression as quantified by PCR (1, 2). This highlights one of the ongoing challenges in the field, namely achieving consensus on standardisation. A higher number of biopsies may be more practical in the large joints focused on in this article. The author notes that there remains a lack of standardisation of techniques for biopsy procedures, and we will later see how this lack of standardisation extends to tissue handling and processing, as well as to reporting in manuscripts. Risks are discussed in general, as well as relevant joint-specific risks, and again these are shown to be acceptable. Lazarou et al. provide a review for US guided biopsy of the small joints, including wrist, MCPs and MTPs, and they also discuss biopsy of a tendon sheath.

Orr et al. describe the technique used for arthroscopically guided synovial biopsies. They provide a comprehensive, step by step explanation of the procedure itself, as well as a discussion regarding the available safety data Orr et al. They describe how arthroscopy represented a pioneering approach in researching inflammatory arthritis, and will continue to complement sonographically guided approaches.

Another major advancement in synovial tissue research has been the increasing accessibility in facilitating the sampling itself. When compared to what has been considered the gold standard procedure for retrieved samples, arthroscopy, US guided procedures have been shown to be less expensive, to yield similar quality tissue, to require less training, and to be more suitable to biopsy small joints (often preferentially involved in rheumatoid arthritis). Humby provides a historical framework to the field, beginning with the first “blind” biopsies by Forestier in the 1930's. The paper discusses in detail the differences between tissue retrieved from arthroscopically guided, as well as the two US guided techniques, outlining the advantages and challenges associated with each. Although there remains robust debate, depending on the quantity of tissue required, whether obtaining lining layer is important, the clinical or research question being addressed, and the joint involved, there is broad consensus that either approach is acceptable in the clinical or research setting. In addition, the attention of the reader is once again drawn to the many studies confirming acceptable safety and tolerability, regardless of technique. Humby also introduces two major international multicentre clinical trials, R4RA and Stratification of Biologic Therapies for RA by Pathobiology, each investigating the role of synovial biopsy in realising precision medicine for rheumatoid arthritis (RA).

Smits et al. discuss the current landscape of synovial sampling to describe to what extent clinical implementation is possible today. They identify that the successful acquisition of synovial tissue is operator dependant, and that although it is obvious that skills need to be retained by regular performance, no minimum requirement in this respect is currently known. They delve into the issues regarding quality assurance and standardisation in all respects of synovial biopsy research, which have been alluded to above. These matters have been the subject of recent intensive and ongoing attempts to harmonise. Synovial biopsy is rarely used in the differential diagnosis of inflammatory arthritis, and it is not entirely clear in which circumstances a synovial biopsy may aid in diagnosis. One definite sub-group that may benefit are those where infective causes are high on the differential, but the synovial fluid has not revealed an organism, or where a non-inflammatory cause for synovial hyperplasia is being considered. The review concludes by articulating the barriers to advancing to more widespread clinical implementation, including in the first instance, making a determination as to what the best quality control to ensure that synovial instead of other joint tissue is acquired.

Manzo et al. also consider the current role of synovial biopsy in the clinics, but they also look towards the objective of being able to stratify individuals with inflammatory arthritis both within and across varying diseases. The authors explain, “One of the most compelling working hypothesis is that the cellular/molecular patho-biology of the inflamed synovial membrane might delineate specific discriminative traits able to improve early diagnosis of undifferentiated forms and patients' stratification into treatment-specific response groups…If differences in the synovial characteristics can be captured between different clinical entities, a cutting-edge question is whether clinically relevant differences can be reliably distinguished also within the same disease, a fundamental premise to conceive the possible integration of synovial biopsy into a precision medicine algorithm.”

While Manzo et al. articulate the hopes for synovial biopsy research to pave a way forward to precision medicine for those with inflammatory arthritis, they also provide an honest account of where we currently are and what challenges remain. There are some “circumstantial” data currently available to support the use of synovial biopsy in the inflammatory arthritis clinical setting, but the discriminative power of this tool to accurately diagnose and prognosticate, as well as to point to a candidate target, remains unproven. Furthermore, they suggest that a stratified approach rather than truly precise approach may hold more promise in the short or medium term.

Arguably, the last number of years have seen the most rapid advances in our understanding of synovial pathobiology, and more precisely, our ability to start considering how to stratify within diseases using these novel insights. Initial attempts were made to achieve this using traditional histopathological observation with important clinical correlates. Later the clinical correlates were related to whole tissue gene expression profiling, with both microarrays and next generation RNA sequencing. Laser capture microdissection and technologies for single cell analysis, are starting to contribute to the effort to stratify disease subtypes. Discrete phenotypes recognisable by varying synovial “signatures,” are now well-described.

In relation to these transcriptomic technologies, Carr et al. detail their development and application, with a particular emphasis on studying fibroblast sub-populations in the synovium (3). The authors explain the initial use of PCR to probe for specific gene expression, which requires pre-identified genes. The ability to test for thousands of genes was provided by cDNA microarrays, and this opened the door to more discovery-based, as distinct from hypothesis-based research. Most recently, RNA sequencing technology has been developed, where the entire transcriptome can be analysed, negating the need to examine specific targeted genes. The potential opportunities as well as caveats are discussed. In addition to the high-throughput transcriptomic technology and methods, Carr et al. discuss how advancements in separating cell subtypes within the joint can be combined with RNA sequencing, to give novel insights into pathobiological processes. One obvious benefit to this approach of separating cell subsets from within the joint rather than analysing whole tissue, is the reduction in the potential to miss subtle but important gene expression signatures from important but numerically few cell subtypes, an observation also made by Triaille and Lauwerys.

Undoubtedly one of the most exciting developments in therapeutics for inflammatory arthritis over the last decade has been the advent of agents targeting the IL-17 pathway. Robert and Miossec discuss IL-17 pathobiology in the joint, examining the role of IL-17 in cartilage and joint destruction, neoangiogenesis, and synergistic effects with TNF alpha. The clinical effects of the various targeted IL-17 therapies in clinical trials are discussed, and possible explanations for the conflicting findings are considered. While accepting that there are “mixed” results in the clinic to targeting IL-17 in RA, they suggest that it may be possible to identify a subset of RA patients, possibly through synovial biopsy analysis, for whom IL-17 is a relevant target. Celis et al. review the synovial biopsy observations in psoriatic arthritis, noting the many similarities with RA, but examining what may be learnt from the differences. They also examine the importance of the role of IL-17, and the pathways associated with it.

A significant challenge is the heterogeneity of the RA phenotype, and this too is reflected in studies of the RA synovium. In the contributions of both Ouboussad et al. as well as Triaille and Lauwerys, this heterogeneity, and the relevance of this to RA research and clinical therapeutics is discussed in detail. Ouboussard et al. provide a review of the effects of biologics and targeted therapies on the synovium, as well as examining synovial predictive markers of response to these therapies.

Triaille and Lauwerys discuss the complexity and challenges of synovial biopsy research to date. They identify some of the key limitations of the data thus far available, including the poor stratification of patients enrolled in synovial biopsy studies, the limited numbers of biopsies performed, as well as the use of retrospective material for research. To further complicate interpreting the data so far collected, the authors draw attention to the plasticity of the RA synovium, varying according to disease duration, serological status, treatment, and, most obviously, disease activity. They also call into question the concept of discrete interpretation of the myeloid and lymphoid pathotypes and suggest a degree of interdependence, offering intriguing evidence to support this viewpoint. The authors hypothesise that a poor response to a specific targeted therapy could either represent an absence of that pathway in the inflamed synovium, or simply disease severity, expressed by several active pathways, overwhelming a highly specific, targeted approach to treatment.

An article collection focussing on synovial sampling and its relevance to precision medicine in arthritis has never been more apt. The last number of years have seen significant advances in our knowledge of synovial pathobiology. New technologies and investigative techniques will likely see this advance further. Despite progress to date, significant challenges remain, and the articles assembled here reflect this reality. The community interested in synovial sampling has never been so large, and the efforts to achieve standardisation never so intense. The largest ever international collaborations utilising synovial biopsies are hopefully about to bear fruit. There is much cause for optimism that we really are about to turn the page to precision medicine in arthritis.

## Author Contributions

All authors listed have made a substantial, direct, and intellectual contribution to the work and approved it for publication.

## Conflict of Interest

The authors declare that the research was conducted in the absence of any commercial or financial relationships that could be construed as a potential conflict of interest.

## Publisher's Note

All claims expressed in this article are solely those of the authors and do not necessarily represent those of their affiliated organizations, or those of the publisher, the editors and the reviewers. Any product that may be evaluated in this article, or claim that may be made by its manufacturer, is not guaranteed or endorsed by the publisher.
